# Apical Hypertrophic Cardiomyopathy (Yamaguchi Syndrome) Presenting as Acute Coronary Syndrome Mimic: A Case Report

**DOI:** 10.7759/cureus.107472

**Published:** 2026-04-21

**Authors:** Lokesh Shirbhate

**Affiliations:** 1 Internal Medicine, N.K.P. Salve Institute of Medical Sciences, Nagpur, IND

**Keywords:** acute coronary syndrome, apical hypertrophy cardiomyopathy, chest pain syndrome, giant t-wave inversion, yamaguchi syndrome

## Abstract

Apical hypertrophic cardiomyopathy (ApHCM), or Yamaguchi syndrome, is a morphological variant of hypertrophic cardiomyopathy that frequently mimics acute coronary syndrome (ACS) due to ischemic-type chest pain and characteristic electrocardiographic changes. A 46-year-old woman presented with angina and deep T-wave inversion in precordial leads. Coronary angiography showed normal epicardial arteries. Transthoracic echocardiography revealed localized left ventricular apical hypertrophy with preserved systolic function and the typical "ace-of-spades" cavity configuration, confirming ApHCM. She was managed conservatively with beta-blockers and guideline-directed therapy, with symptomatic improvement. Early recognition prevents unnecessary invasive procedures and facilitates appropriate long-term management.

## Introduction

Apical hypertrophic cardiomyopathy (ApHCM), also known as Yamaguchi syndrome, is a distinct morphological variant of hypertrophic cardiomyopathy characterized by localized hypertrophy predominantly involving the left ventricular apex. First described in Japanese populations by Yamaguchi et al. [1], it is now increasingly recognized worldwide, particularly among Asian cohorts, although its prevalence remains lower in Western populations [2].

Clinically, ApHCM often presents with nonspecific symptoms such as chest pain, dyspnea, palpitations, or syncope. Notably, the chest pain frequently mimics acute coronary syndrome (ACS), both in presentation and in electrocardiographic findings, leading to diagnostic challenges [3]. Electrocardiography typically demonstrates giant negative T-wave inversions, especially in precordial leads, which may be mistaken for ischemic heart disease [4].

The underlying pathophysiology of symptoms in ApHCM is multifactorial and includes microvascular dysfunction, impaired myocardial perfusion, and increased oxygen demand, even in the absence of obstructive coronary artery disease [5]. Consequently, many patients undergo invasive coronary angiography before a definitive diagnosis is established [6].

Echocardiography remains the initial imaging modality of choice, demonstrating characteristic apical hypertrophy and the classic "ace-of-spades" configuration of the left ventricular cavity. Advanced imaging techniques such as cardiac magnetic resonance imaging further enhance diagnostic accuracy and risk stratification by identifying myocardial fibrosis [7,8].

This case highlights the importance of recognizing ApHCM as a differential diagnosis in patients presenting with angina-like symptoms and electrocardiographic abnormalities suggestive of ACS but with normal coronary arteries.

## Case presentation

A 46-year-old woman with no prior history of cardiovascular disease presented to the emergency department with complaints of chest pain of two days' duration. The pain was described as retrosternal, intermittent, and constricting in nature, with occasional radiation to the left precordium. Each episode lasted approximately 10-15 minutes and was not clearly related to exertion. There was no associated history of syncope, presyncope, palpitations, diaphoresis, nausea, or vomiting. She denied any prior similar episodes.

The patient had no known history of hypertension, diabetes mellitus, dyslipidemia, or smoking. There was no significant family history of premature coronary artery disease or sudden cardiac death.

On presentation, the patient was hemodynamically stable with a blood pressure of 120/80 mmHg, a heart rate of 78 beats per minute (regular), a respiratory rate of 16 breaths per minute, and an oxygen saturation of 98% on room air. Cardiovascular examination revealed normal first and second heart sounds with no murmurs, rubs, or gallops. Respiratory, abdominal, and neurological examinations were unremarkable.

**Figure 1:**
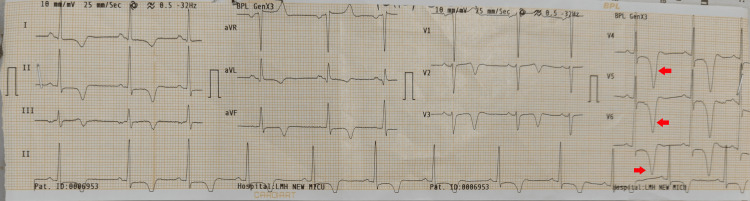
Electrocardiogram demonstrating characteristic T-wave abnormalities in ApHCM The 12-lead electrocardiogram shows diffuse T-wave inversions, with deep, symmetrical negative T waves prominently in leads V4-V6 (indicated by digital arrows). These findings are highly suggestive of ApHCM and may mimic myocardial ischemia or acute coronary syndrome. Such "giant negative T waves" are a well-recognized electrocardiographic hallmark of this condition [1,5]. ApHCM: apical hypertrophic cardiomyopathy

Given the clinical suspicion of ACS, a 12-lead electrocardiogram (ECG) was performed, which demonstrated global T-wave inversions across multiple leads, with deep, symmetrical T-wave inversions most prominent in the precordial leads V4-V6. There was no ST-segment elevation or pathological Q waves (Figure 1). Serial cardiac biomarkers, including troponin I, were within normal limits.

In view of persistent symptoms and concerning electrocardiographic findings, the patient underwent urgent coronary angiography. The angiogram revealed normal epicardial coronary arteries with no evidence of atherosclerotic disease, luminal narrowing, or coronary anomalies.

**Figure 2:**
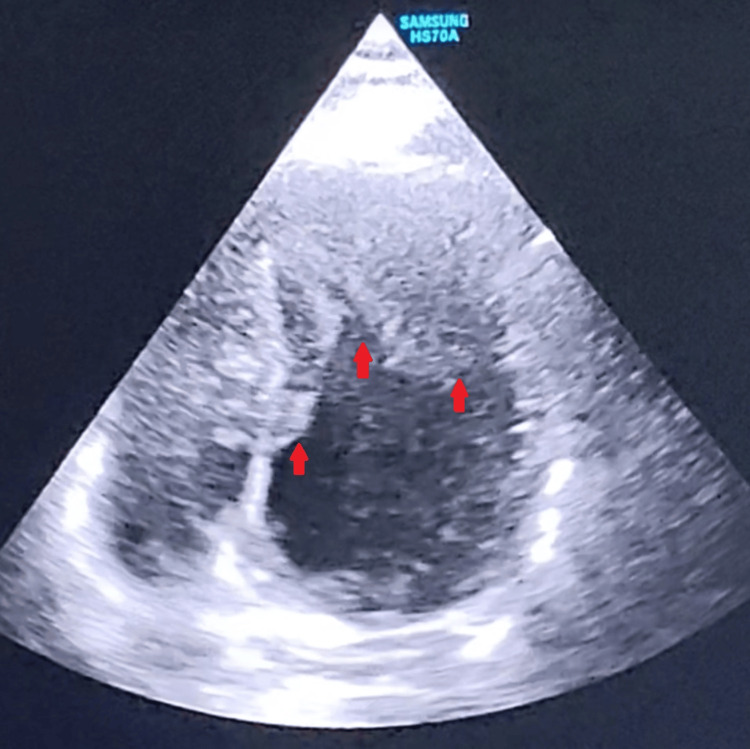
Transthoracic echocardiography showing localized apical myocardial hypertrophy Two-dimensional echocardiographic imaging demonstrates asymmetric hypertrophy confined to the left ventricular apex, with preserved thickness of basal segments. Digital outline highlights the region of maximal apical wall thickening. This pattern is characteristic of ApHCM and helps differentiate it from other forms of hypertrophic cardiomyopathy [6]. ApHCM: apical hypertrophic cardiomyopathy

Subsequently, transthoracic echocardiography was performed for further evaluation. Two-dimensional imaging revealed localized hypertrophy confined to the left ventricular apex, with maximal wall thickness noted at the apical segments. The basal and mid-ventricular segments were of normal thickness. There was no evidence of left ventricular outflow tract obstruction at rest. Global left ventricular systolic function was preserved, with an estimated ejection fraction of approximately 60% (Figure 2, Video 1, and Video 2).

**Video 1:** PLAX view demonstrating ApHCM This transthoracic echocardiographic PLAX view demonstrates increased myocardial thickness toward the left ventricular apex, with preserved basal segment thickness and overall systolic function. Although apical visualization may be limited in this view, the findings support asymmetric hypertrophy consistent with ApHCM. Such structural abnormalities are best appreciated in combination with other echocardiographic views [6]. PLAX: parasternal long-axis; ApHCM: apical hypertrophic cardiomyopathy

**Video 2:** Apical four-chamber view (zoomed) demonstrating left ventricular apical hypertrophy This zoomed apical four-chamber view provides a focused assessment of the left and right ventricles, clearly demonstrating localized hypertrophy of the left ventricular apex with relative preservation of the right ventricle. The left ventricular cavity appears narrowed toward the apex, consistent with ApHCM. This view enhances the visualization of apical morphology and helps for confirming the diagnosis. ApHCM: apical hypertrophic cardiomyopathy

**Figure 3:**
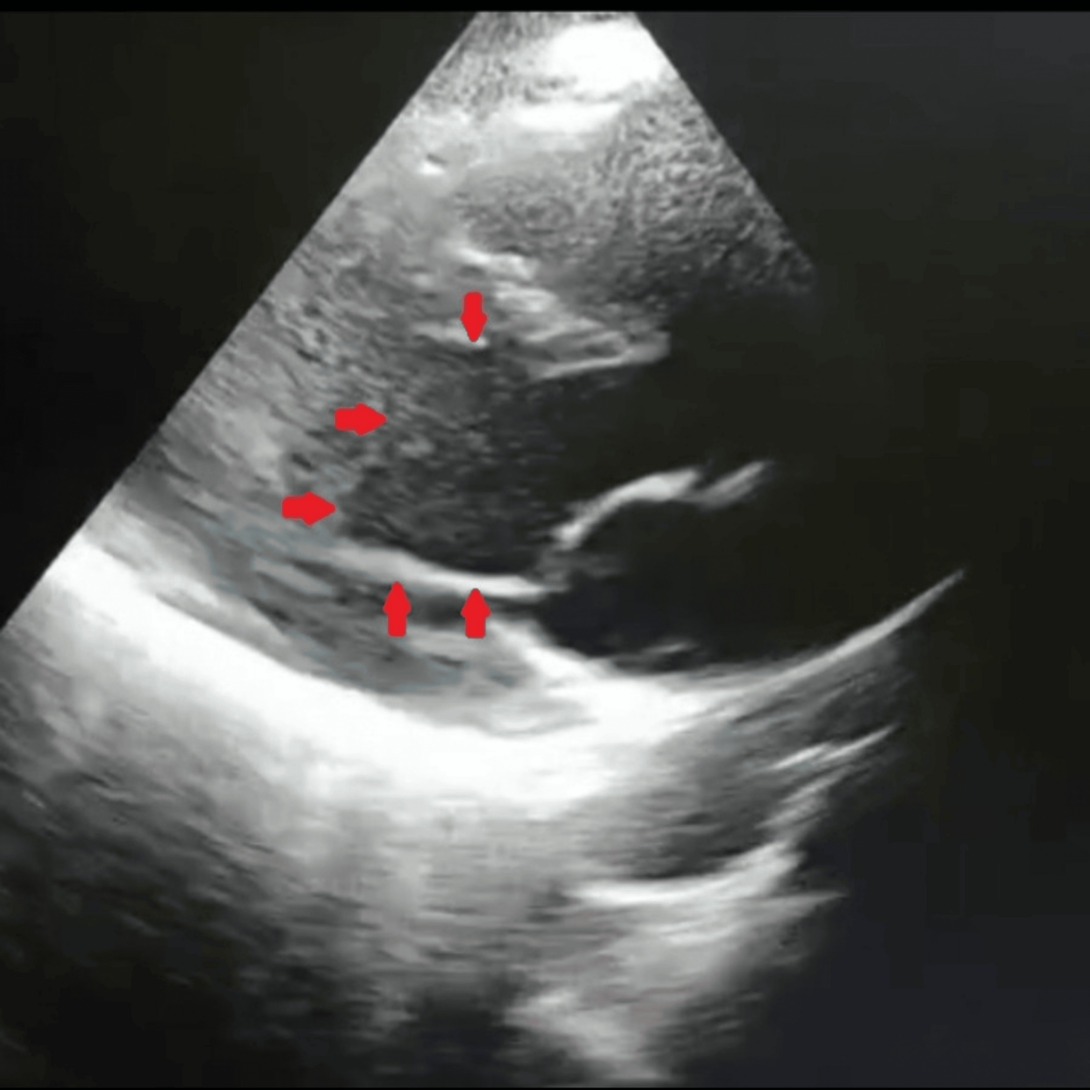
Echocardiographic "ace-of-spades" configuration of the left ventricular cavity PLAX view during diastole reveals the classic "ace-of-spades" morphology of the left ventricular cavity, resulting from apical hypertrophy and cavity obliteration. The digital circle highlights the narrowed apical cavity, a key diagnostic feature of ApHCM [6,7]. PLAX: parasternal long-axis; ApHCM: apical hypertrophic cardiomyopathy

Notably, the left ventricular cavity exhibited the characteristic "ace-of-spades" configuration during end-diastole in parasternal long-axis views, due to apical cavity obliteration. There was no significant mitral regurgitation or systolic anterior motion of the mitral valve (Figure 3, Video 1, and Video 2).

These imaging findings, in conjunction with the electrocardiographic pattern of deep T-wave inversions and the absence of obstructive coronary artery disease, were diagnostic of ApHCM (Yamaguchi syndrome).

## Discussion

Clinical background

ApHCM, commonly referred to as Yamaguchi syndrome, represents a morphologically distinct subtype of hypertrophic cardiomyopathy in which myocardial thickening predominantly involves the left ventricular apex. Since its initial characterization in 1976, this entity has been reported with higher frequency in East Asian populations, whereas it remains relatively uncommon in Western cohorts [1,2].

Clinical presentation and biomarker significance

Patients with ApHCM frequently present with symptoms that resemble ACS, most notably chest pain. The underlying mechanism is not attributable to epicardial coronary obstruction but is thought to involve a combination of impaired coronary microcirculation, increased myocardial oxygen demand, and elevated intracavitary pressures leading to subendocardial ischemia [3]. Consequently, individuals often undergo invasive coronary evaluation before a definitive diagnosis is established, as occurred in the present case [4].

Electrocardiographic abnormalities are a prominent feature and may provide an important diagnostic clue. Deep, symmetric T-wave inversions, particularly in the precordial leads, are frequently observed and may be marked in severity. Although these findings are not exclusive to ApHCM, their presence in the appropriate clinical setting should prompt the consideration of this diagnosis, especially when cardiac biomarkers are not elevated [1,5]. Cardiac troponin elevation in hypertrophic cardiomyopathy may occur even in the absence of obstructive coronary artery disease due to microvascular ischemia, increased myocardial oxygen demand, elevated wall stress, and ongoing myocyte injury. Elevated troponin may reflect active myocardial damage beyond structural abnormalities seen on imaging. Mild persistent elevation usually indicates chronic low-grade injury, whereas higher or rising levels may suggest more advanced disease. Higher high-sensitivity troponin levels have been associated with myocardial fibrosis, greater wall thickness, and poorer cardiovascular outcomes, making serial measurement useful for prognostic assessment [6].

Diagnostic evaluation

Echocardiography remains the initial imaging modality of choice and typically demonstrates localized hypertrophy confined to the apical segments, with preservation of basal myocardial thickness. A characteristic morphological feature is the spade-like configuration of the left ventricular cavity during diastole, resulting from apical cavity narrowing. This finding was clearly evident in our patient and provided a key diagnostic indicator [7]. However, due to limitations in acoustic window and apical visualization, echocardiography may occasionally underestimate the extent of hypertrophy.

Cardiac magnetic resonance imaging offers improved delineation of myocardial architecture and allows more precise quantification of wall thickness. In addition, the identification of late gadolinium enhancement enables the assessment of myocardial fibrosis, which has been associated with an increased risk of arrhythmic events and adverse clinical outcomes [8].

Differential diagnosis

The differential diagnosis includes ACS, Wellens syndrome, Takotsubo cardiomyopathy, and myocarditis, all of which may present with chest pain and marked T-wave inversion. Wellens syndrome typically reflects critical proximal left anterior descending artery stenosis, while Takotsubo cardiomyopathy demonstrates transient apical ballooning with reversible systolic dysfunction. Myocarditis may present with biomarker elevation and myocardial edema on advanced imaging. In contrast, persistent apical hypertrophy with the characteristic "ace-of-spades" left ventricular configuration and normal coronary arteries favored the diagnosis of ApHCM in our patient [9].

Prognosis and complications

Although ApHCM has historically been regarded as a relatively benign form of hypertrophic cardiomyopathy, accumulating data suggest that it may be associated with clinically significant complications. These include the development of apical aneurysms, ventricular arrhythmias, thromboembolic events, and, in rare instances, sudden cardiac death [10]. Therefore, careful clinical assessment and periodic follow-up are warranted.

Additional observational studies have shown that atrial fibrillation, apical myocardial infarction, and progressive heart failure may also occur during long-term follow-up, even in patients who initially appear clinically stable. Female sex and coexisting atrial fibrillation have been associated with less favorable outcomes in some retrospective analyses, emphasizing that the long-term course may not always be benign [11].

Genetic considerations and secondary prevention

Mutations in sarcomeric protein genes such as MYH7 and MYBPC3 have been described in some patients with ApHCM, although the genetic basis of the apical phenotype remains less clearly defined than that of classical hypertrophic cardiomyopathy. Additional sarcomeric and cardiac actin-related variants have also been reported in selected cases, further supporting a heterogeneous genetic background. In patients with a family history of cardiomyopathy or sudden cardiac death, genetic testing may be considered, and screening of first-degree relatives can facilitate the early identification of subclinical disease. However, because risk prediction models specific to ApHCM remain limited, management decisions, including consideration of implantable cardioverter-defibrillator implantation, should be individualized based on conventional high-risk markers and overall clinical assessment. Continued long-term follow-up is essential, as complications such as atrial fibrillation, apical aneurysm formation, thromboembolic events, and progressive ventricular dysfunction may develop over time even in initially stable patients [11].

Management

Management is largely guided by symptom burden and risk stratification. Pharmacological therapy with beta-adrenergic blockers or non-dihydropyridine calcium channel blockers is commonly employed to improve diastolic relaxation and reduce myocardial oxygen demand. Anticoagulation should be considered in patients with atrial fibrillation or evidence of thrombus formation. Selected high-risk individuals may require device-based therapy, including implantable cardioverter-defibrillators, for the primary or secondary prevention of sudden cardiac death [12].

Relevance of the present case

In the present case, the diagnosis was particularly challenging because the patient presented with ischemic-type chest pain and marked electrocardiographic abnormalities strongly suggestive of ACS, despite persistently normal cardiac biomarkers. The absence of obstructive coronary artery disease on angiography necessitated further evaluation, which subsequently demonstrated localized apical hypertrophy with the characteristic left ventricular cavity morphology on transthoracic echocardiography. The unusual aspect of this case lies in the close clinical resemblance to acute ischemic heart disease in a patient without conventional cardiovascular risk factors, illustrating how ApHCM may remain unrecognized unless specifically considered during diagnostic evaluation.

## Conclusions

ApHCM (Yamaguchi syndrome) is an important and often under-recognized cause of angina-like chest pain associated with striking electrocardiographic abnormalities, frequently mimicking ACS. This case underscores the need for a high index of clinical suspicion, particularly in patients demonstrating deep precordial T-wave inversions in the absence of biomarker elevation or obstructive coronary artery disease. Transthoracic echocardiography plays a pivotal role in establishing the diagnosis by identifying localized apical hypertrophy and the characteristic "ace-of-spades" configuration of the left ventricular cavity, while advanced imaging modalities such as cardiac magnetic resonance imaging may further aid in risk stratification. Although traditionally considered a relatively benign entity, ApHCM carries a potential risk of adverse cardiovascular events, including arrhythmias and thromboembolic complications, necessitating appropriate clinical follow-up. Early recognition is essential to avoid misdiagnosis, prevent unnecessary interventions, and guide optimal long-term management.
